# A Novel Role of Dickkopf-Related Protein 3 in Macropinocytosis in Human Bladder Cancer T24 Cells

**DOI:** 10.3390/ijms17111846

**Published:** 2016-11-05

**Authors:** Nonoka Tsujimura, Nami O. Yamada, Yuki Kuranaga, Minami Kumazaki, Haruka Shinohara, Kohei Taniguchi, Yukihiro Akao

**Affiliations:** 1 United Graduate School of Drug Discovery and Medical Information Sciences, Gifu University, 1-1 Yanagido, Gifu-city, Gifu 501-1194, Japan; nono.pi306@gmail.com (N.T.); v3501001@edu.gifu-u.ac.jp (Y.K.); t3501001@edu.gifu-u.ac.jp (M.K.); harukashinohara313@gmail.com (H.S.); sur144@osaka-med.ac.jp (K.T.); yakao@gifu-u.ac.jp (Y.A.); 2 Department of Anatomy, Graduate School of Medicine, Gifu University, 1-1 Yanagido, Gifu-city, Gifu 501-1194, Japan

**Keywords:** Dickkopf-3, macropinocytosis, autophagy, nutrition

## Abstract

Dickkopf-related protein 3 (Dkk-3) is a potential tumor suppressor reported in various cancer entities. However, we found that Dkk-3 was exceptionally upregulated in bladder cancer T24 cells. To validate the biological role of Dkk-3 other than a tumor suppressor, we examined the function of Dkk-3 in T24 cells. Gene silencing of Dkk-3 inhibited cell growth through inducing G_0_/G_1_ cell-cycle arrest. Furthermore, Dkk-3 knock-down caused macropinocytosis accompanied by autophagy, which were canceled in part by their inhibitors 5-(*N*-ethyl-*N*-isopropyl) amiloride (EIPA) and 3-methyladenine (3-MA). The macropinocytosis was induced by the Dkk-3 knock-down when there were sufficient extracellular nutrients. On the other hand, when the nutritional condition was poor, the autophagy was mainly induced by the Dkk-3 knock-down. These data indicated that Dkk-3 has a role in modulating macropinocytotic and autophagic pathways, a distinct function other than a Wnt antagonist.

## 1. Introduction

The Wnt signaling pathway regulates cell proliferation, differentiation, fate and death in multiple biological processes ranging from embryonic development to cancer progression [1]. Well-characterized Wnt signaling is Wnt/β-catenin signaling; Wnt ligand binds to a frizzled (Fz), recruits low-density lipoprotein receptor-related proteins 5 and 6 (LRP5/6) to the Fz, induces the formation of the receptor complex of Fz/LRP5/6 and activates downstream signal transduction via β-catenin [2]. The Dickkopf-related protein (Dkk) family is known as a secreted Wnt antagonist. Secreted Dkk binds to the LRP5/6, disrupts the formation of the Fz/LRP5/6 complex and consequently inhibits Wnt/β-catenin signaling. Dkk-1, -2 and -4 share the inhibitory effect on Wnt/β-catenin signaling. However, it has been shown that Dkk-3 does not bind to LRP5/6 or affect Wnt/β-catenin signaling [3]. So far, no specific receptor for Dkk-3 has been identified. On the other hand, some studies have demonstrated that Dkk-3 can antagonize Wnt and inhibit Wnt/β-catenin signaling [4,5,6]. Thus, the role of secreted Dkk-3 in the Wnt/β-catenin signaling is still elusive. Dkk-3 is also called REIC (reduced expression in immortalized cells), because its endogenous expression is decreased in the immortalized cells [7]. Downregulation of endogenous Dkk-3/REIC is frequently observed in most cancers. Overexpression of Dkk-3 can induce apoptosis via c-jun N-terminal kinase (JNK) activation in prostate cancer [8] and non-small cell lung cancer [9], via the mitochondrial pathway in colorectal cancer [10] and mucinous ovarian cancer [11], by obstructing β2-microglobulin-mediated VGFR-2/Akt/mTOR signaling in ovarian cancer [12] or enhancing the anti-tumor effect of gemcitabine in pancreatic cancer [13]. These studies have concluded that Dkk-3 is an effective tumor suppressor and potential target in cancer gene therapy [14]. Significant involvements of Dkk-3 in tumor angiogenesis have also been reported [15]. Recently, we have validated that *Dkk-3* is one of the direct targets of microRNA-92a, and downregulation of Dkk-3 can promote angiogenesis in endothelial cells [16]. However, a precise role of Dkk-3 in carcinogenesis remains to be validated. We found that Dkk-3 is exceptionally overexpressed in T24 cells, and therefore, we focused on T24 cells to validate the function of endogenous Dkk-3 in this study. As a novel function of Dkk-3 was speculated, we investigated the effects of Dkk-3 knock-down by RNA interference on cell morphology, proliferation and intracellular signaling in T24 cells. We finally found that Dkk-3 has a novel role in modulating both macropinocytotic and autophagic pathways.

## 2. Results

### 2.1. Dkk-3 Expression Was Specifically Upregulated in Human Bladder Cancer T24 Cells

**Figure 1:**
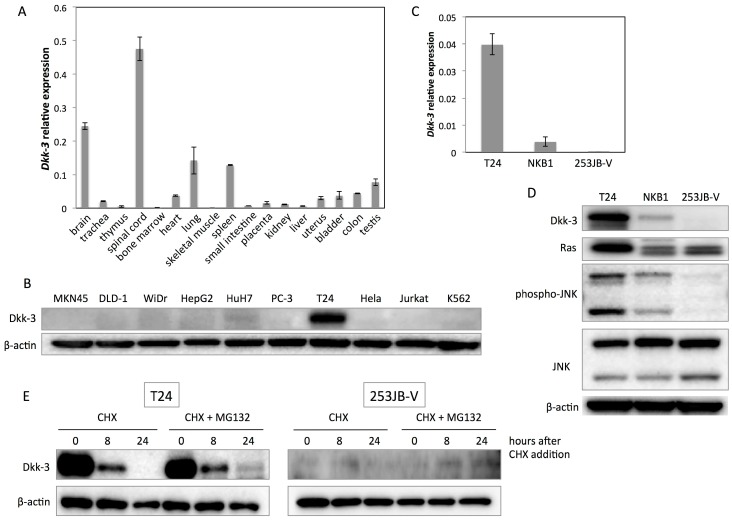
Specific overexpression of Dkk-3 in T24 cells. (**A**) Relative expression levels of Dkk-3 mRNA in various human normal tissues. The expression levels of Dkk-3 mRNA were normalized by those of GAPDH mRNA; (**B**) Dkk-3 protein expressions in various human cancer cell lines; (**C**) Relative expression levels of Dkk-3 mRNA in human bladder cancer cell lines, T24, NKB1 and 253J-BV; (**D**) Protein expression profiles of the bladder cancer cell lines; (**E**) The stability of Dkk-3 protein in T24 and 253JB-V cells determined by treatment with 300 μg/mL cycloheximide (CHX) in the presence and absence of MG132 (5 μM). DMSO (5 μM) was used as negative control reagent for MG132.

Firstly, we examined the tissue distribution of Dkk-3 mRNA and found that the endogenous expression levels of Dkk-3 mRNA in brain and spinal cord tissues were relatively high compared with those in the other tissues tested (Figure 1A). Second, to evaluate the Dkk-3 protein expression levels in various kinds of cancer cell lines, we performed Western blot analysis (Figure 1B). As a result, Dkk-3 protein expression was especially upregulated in human bladder cancer T24 cells. Furthermore, we evaluated Dkk-3 protein expression levels in other human bladder cancer cell lines (NKB1 and 253JB-V) and found that specific upregulation of Dkk-3 in T24 cells (Figure 1C,D). JNK activation and overexpression of Ras were also detected in T24 cells (Figure 1D). We also examined the stability of Dkk-3 protein by using cycloheximide (CHX), an inhibitor for protein synthesis at the translational step, and MG132, an inhibitor for proteasome. Dkk-3 protein in both T24 and 253JB-V cells was degraded at 24 h after the treatment with CHX (Figure 1E). To verify whether Dkk-3 degradation occurs via the proteasome, we also co-treated cells with CHX and MG132 to inhibit proteasomal degradation. MG132 treatment partly inhibited the degradation of Dkk-3 protein in both T24 and 253JB-V cells (Figure 1E).

### 2.2. Dkk-3 Acted as a Growth-Related Molecule in T24 Cells

**Figure 2:**
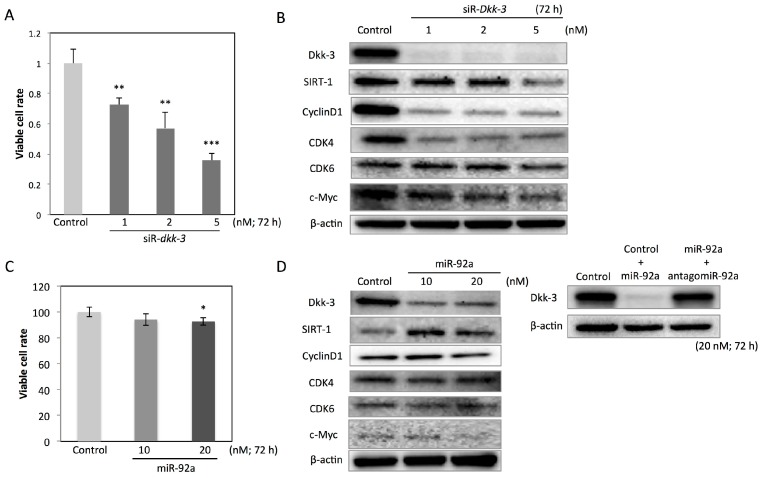

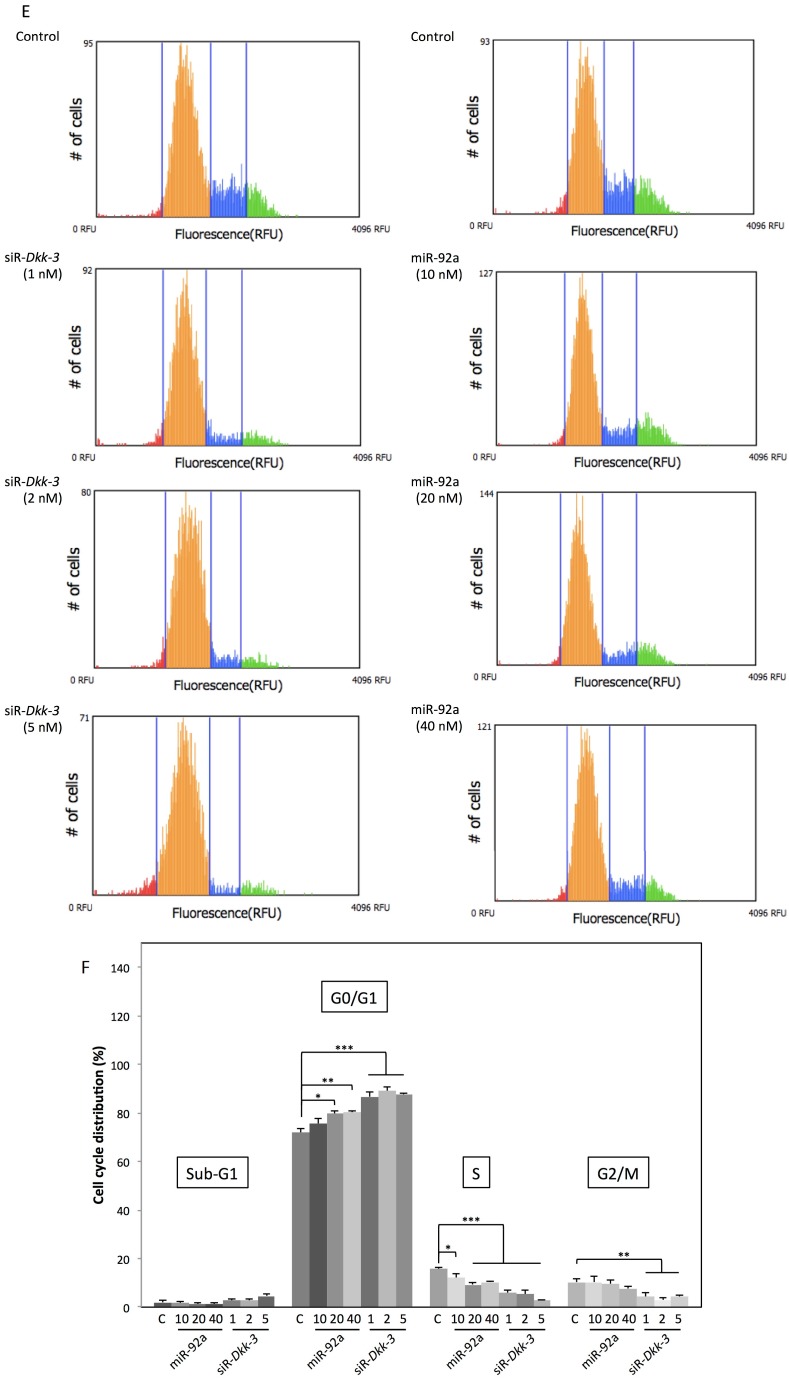

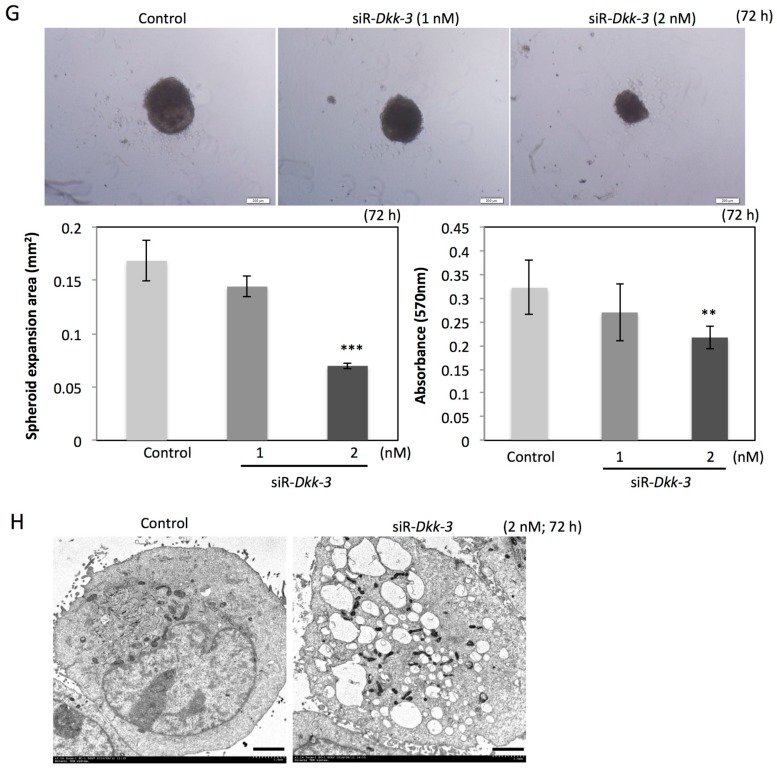
Anti-oncogenic effects of Dkk-3 knock-down by using siR-*Dkk-3* or miR-92a in T24 cells. (**A**,**B**) Cell viability (**A**) and protein expression profiles (**B**) in T24 cells at 72 h after transfection with non-specific siRNA or siR-*Dkk-3* (1, 2 or 5 nM); (**C**,**D**) Cell viability (**C**) and protein expression profiles (**D**) in T24 cells at 72 h after transfection with non-specific siRNA or miR-92a (10 or 20 nM). Dkk-3 expression also examined in T24 cells transfected with non-specific siRNA (20 nM), non-specific siRNA (10 nM) + miR-92a (10 nM) or miR-92a (10 nM) + antagomiR-92a (10 nM); (**E**,**F**) Cell-cycle distribution of T24 cells at 72 h after transfection with non-specific siRNA, siR-*Dkk-3* or miR-92a. Cells were classified into 4 phases; sub-G1 phase (red), G0/G1 phase (yellow), S phase (blue), and G2/M phase (green); (**G**) 3D spheroid colorimetric proliferation/viability assay in T24 cells at 72 h after the spheroid formation. The spheroid expansion area was calculated according to the manufacturer’s protocol. Absorbance at 570 nm means viability of the spheroid assessed by the MTT assay. Scale bars, 200 μm; (**H**) Electron microscopic observation of T24 cells at 72 h after transfection with non-specific siRNA or siR-*Dkk-3.* Scale bars, 2 μm. The *p*-values in (**A**,**C**,**F**,**G**) are indicated as follows: * *p* < 0.05, ** *p* < 0.01 and *** *p* < 0.001.

Next, we knock-downed *Dkk-3* in T24 cells and evaluated its effects. Silencing *Dkk-3* by siR-*Dkk-3* significantly decreased the viable cell rate at 72 h after the transfection (Figure 2A). We further examined the mechanism underling growth inhibition caused by silencing *Dkk-3*. *Dkk-3* knock-down led to the downregulation of sirtuin1 (SIRT1) and cell-cycle promoter proteins, including cyclinD1, CDK4, CDK6 and c-Myc (Figure 2B). Previously, we reported that *Dkk-3* is one of the target genes for miR-92a regulation in colorectal cancer cells [16]. The ectopic expression of miR-92a in T24 cells also downregulated Dkk-3, and combined transfection with miR-92a and antagomiR-92a restored the expression level of Dkk-3 (Figure 2D). However, the inhibitory effect on growth by the ectopic expression of miR-92a was weak, and SIRT1 expression was increased in T24 cells transfected with miR-92a (Figure 2C,D). Analysis of the cell-cycle distribution revealed the G_0_/G_1_ arrest in T24 cells transfected with siR-*Dkk-3*, which was severer than that in the cells transfected with miR-92a (Figure 2E,F). The 3D spheroid colorimetric viability assay showed that *Dkk-3* knock-down significantly inhibited expansion of T24 cell-derived spheroids (Figure 2G). To further evaluate the effects of *Dkk-3* knock-down on T24 cells, we observed the cells transfected with siR-*Dkk-3* by electron microscopy. *Dkk-3* knock-down caused the accumulation of single membranous vesicular structures containing fluids in the cytoplasm (Figure 2H).

### 2.3. Dkk-3 Knock-Down Caused Macropinocytosis Corresponding to the Extracellular Nutritional Condition

**Figure 3:**
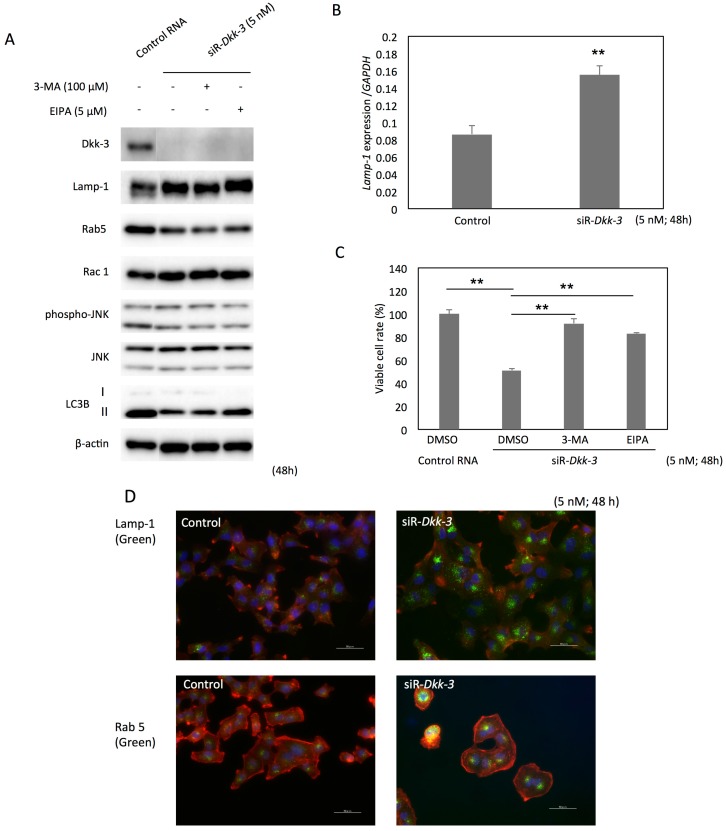

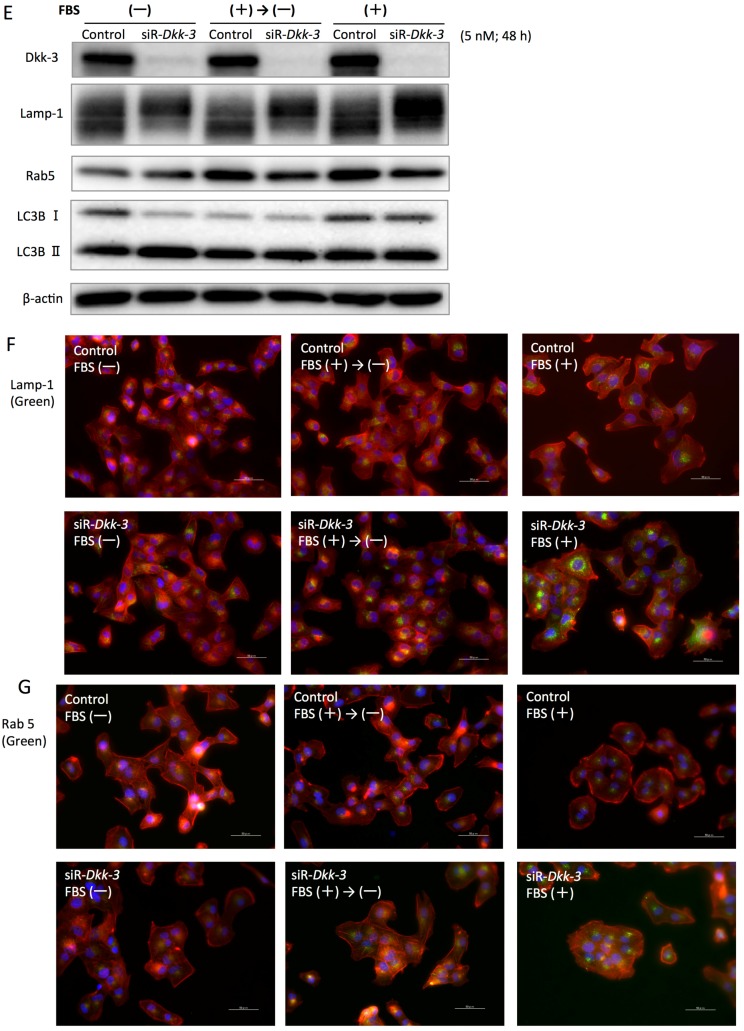
*Dkk-3* knock-down induces macropinocytosis and autophagy depending on the extracellular nutritional condition in T24 cells. (**A**) Expression profiles of macropinocytosis or autophagy-related proteins in T24 cells at 48 h after transfection with non-specific siRNA or siR-*Dkk-3*; (**B**) The mRNA level of Lamp-1 at 48 h after the transfection with siR-*Dkk-3*; (**C**) Cell viability at 48 h after the transfection with siR-*Dkk-3* or non-specific siRNA. Cells were co-treated with DMSO, macropinocytosis inhibitor EIPA or autophagy inhibitor 3-MA (5 nM for 48 h); (**D**) Immunocytochemistry of T24 cells at 48 h after the transfection with non-specific siRNA or siR-*Dkk-3* (5 nM). Lamp-1 (green), Rab5 (green), F-actin (red) and the nuclei (blue) are shown. Scale bars, 50 μm; (**E**) Switching between autophagy and macropinocytosis in the siR-*Dkk-3* transfected T24 cells depending on the extracellular nutritional condition. T24 cells were maintained in three different nutritional conditions; FBS was deprived for 48 h (−); FBS was added for 24 h and then deprived for 24 h (+) → (−); or FBS was added for 48 h (+) after the transfection (5 nM); (**F**,**G**) Immunocytochemistry of T24 cells at 48 h after the transfection with non-specific siRNA or siR-*Dkk-3* in the three different nutritional conditions mentioned above*.* Scale bars, 50 μm. The *p*-values in (**B**,**C**) are indicated as follow: ** *p* < 0.01.

The morphological observation by electron microscopy revealed that the accumulated vesicular structures were different from autophagosomes or apoptotic bodies (Figure 2H). Western blot analysis at 72 h after the siR-*Dkk-3* transfection detected an increase in the 42-kDa fragment of PARP-1, but not conversion of LC3B-I to LC3B-II (Figure S1). Lamp-1, a lysosomal membrane protein, was upregulated, while Rab5, an early endosomal component, was downregulated at 48 h after the *Dkk-3* knock-down (Figure 3A). As to Lamp-1, the level of Lamp-1 mRNA was also elevated after *Dkk-3* knock-down (Figure 3B). The Wnt-Rac1-JNK axis was inactivated, whereas the p38 and Erk1/2 pathways were activated (Figure S1). These results indicate that the *Dkk-3* knock-down stimulated a kind of endocytic process, morphologically considered to be a macropinocytosis, which represents a biologically important tool for the up-take of extracellular nutrients. Then, we explored the functional assay using a macropinocytosis inhibitor, 5-(*N*-ethyl-*N*-isopropyl) amiloride (EIPA), and an autophagy inhibitor, 3-methyladenine (3-MA), to determine the machinery of the vesicle formation induced by the *Dkk-3* knock-down. As a result, treatment with each inhibitor at the indicated concentration restored the viable cell rate at 48 h after the transfection with siR-*Dkk-3* (Figure 3C). Morphologically, the vacuoles in the cells transfected with siR-*Dkk-3* almost disappeared in the EIPA-treated cells (data not shown). Immunocytochemical staining showed that Lamp-1 and Rab5 were accumulated around the nucleus in the cells transfected with siR-*Dkk-3* (Figure 3D). When extracellular nutrients were deprived, the *Dkk-3* knock-down led to the activation of autophagic process, confirmed by the increased conversion of LC3B-I to LC3B-II (Figure 3E). However, when extracellular nutrients existed, Lamp-1 was upregulated, and the conversion of LC3B-I to LC3B-II was not observed (Figure 3E). Rab5 was upregulated according to the addition of nutrients (Figure 3F,G).

### 2.4. Dkk-3 Overexpression Partially Canceled the Growth Inhibitory Effect and Macropinocytosis Induction of siR-Dkk-3 on T24 Cells

**Figure 4:**
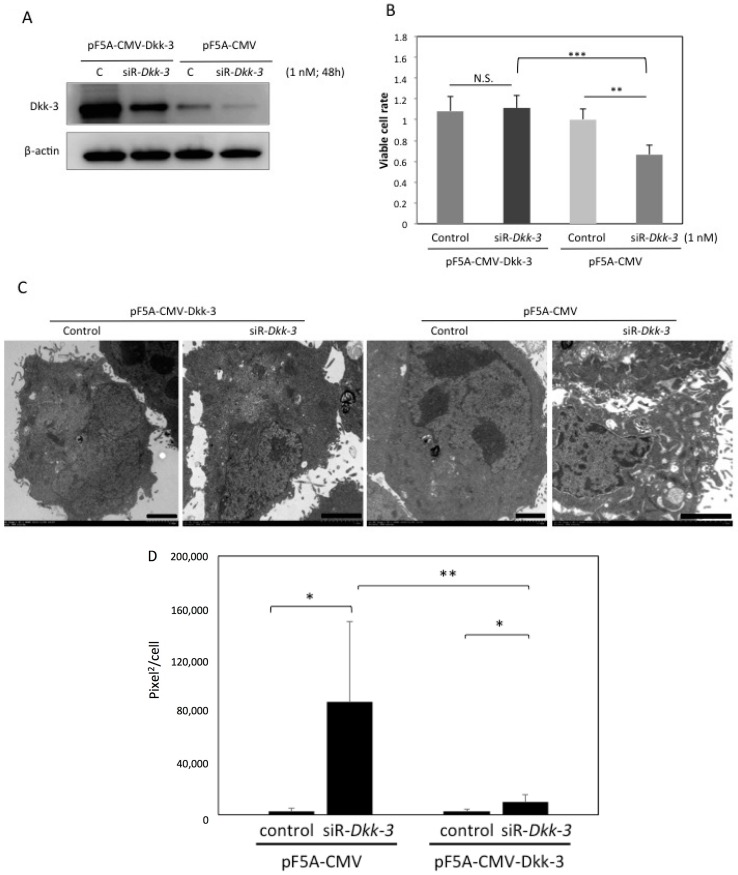
Dkk-3 overexpression diminished the inhibitory effects of siR-*Dkk-3* on T24 cells. (**A**) Dkk-3 expression examined at 48 h after the co-transfection with the pF5A-CMV-Dkk-3 vector (0.5 μg/mL) and non-specific siRNA or siR-*Dkk-3* (1 nM). The pF5A-CMV vector was used as the control vector; (**B**) Cell viability and (**C**) electron microscopic observation of T24 cells at 48 h after the transfection. Scale bars, 2 μm; (**D**) The area of macropinocytotic vesicles in each cell was measured by ImageJ. The *p*-values in (**B**,**D**) are indicated as follows: N.S., not significant; * *p* < 0.05, ** *p* < 0.01 and *** *p* < 0.001.

If Dkk-3 were indispensable to suppress the macropinocytotic process, overexpression of Dkk-3 could cancel the effects of siR-*Dkk-3* on T24 cells. Dkk-3 was transiently overexpressed in T24 cells by using the pF5A-CMV-Dkk-3 vector. Dkk-3 overexpression diminished the inhibitory effects of siR-*Dkk-3* on the viable cell rate and Dkk-3 protein expression in T24 cells (Figure 4A,B). Electron microscopic observation also confirmed that macropinocytosis caused by the *Dkk-3* knock-down was markedly reduced by the Dkk-3 overexpression (Figure 4C). Macropinocytosis was quantified by calculating the vesicular area within the cells observed by electron microscopy (Figure 4D).

## 3. Discussion

Endogenous Dkk-3 is frequently downregulated in cancers and has been studied as a potential anti-oncogene [17,18]. In our study, we found that Dkk-3 was exceptionally upregulated in bladder cancer T24 cells. Therefore, we considered T24 cells as a key to validate a novel function of Dkk-3. We did not find the genomic amplification of the *Dkk-3* coding region in T24 cells (data not shown). The stability of Dkk-3 protein was also investigated by using cycloheximide (CHX). N-terminal residues of Dkk-3 are MQ (methionine-glutamine), which are considered to be a target of the Arg/N-end rule pathway [19]. The N-end rule pathway recognizes specific N-terminal residues and ubiquitinates the proteins for proteasomal degradation [20]. If T24 cells had a mutation or modification, including phosphorylation in the N-terminal residues of the Dkk-3 protein, degradation of the Dkk-3 protein may delay and lead to its specific overexpression in T24 cells [21]. However, our data demonstrated that Dkk-3 protein in T24 cells was degraded by the proteasome as observed in 253JB-V cells. Thus, the stability of Dkk-3 protein in T24 cells was not altered, and the cause of the specific overexpression of Dkk-3 cannot be attributable to the protein stability.

T24 cells displayed a large and flat morphology and became positive for the β-Gal staining after the *Dkk-3* knock-down (Figure S3). The *Dkk-3* knock-down also downregulated SIRT1 and induced cell-cycle arrest. Therefore, we first considered that Dkk-3 has a role in the senescence induction. We further explored the Rb pathway; however, T24 cells did not express p16/Rb and, consequently, our molecular examination of the role of Dkk-3 in the senescence induction was pending.

We observed the senescence-like T24 cells by using electron microscopy. The cells were full of membranous vesicular structures containing extracellular fluids. Morphologically, apoptosis and necrosis were denied. Various studies reported similar morphological observations called macropinocytosis, an endocytotic process previously considered to be a subtype of autophagy [22,23]. The mechanism of macropinocytosis is similar to that of phagocytosis. The ruffled plasma membrane extends to encompass extracellular fluids. The fluids are internalized according to the fusion of the membranous protrusions by themselves [24]. Ras, PI3K, Src and Rac1 activities have been shown to stimulate macropinocytosis [25,26,27,28,29]. Since Ras was upregulated in T24 cells in this study, it is possible that the Ras upregulation coordinately stimulated macropinocytosis with Dkk-3 and Rab5 [30]. Rab5 localizes in the early macropinosomes, and Lamp-1 acquisition occurs according to the fusion of macropinosomes with lysosomes. It has been reported that overexpression of Rab5 increases the number of macropinosomes and stimulates fluid up-take, whereas the downregulation of Rab5 inhibits these processes [30]. In the immunocytochemical analysis, our data demonstrated that *Dkk-3* knock-down strengthened the staining intensity of Rab5; however, Western blot analysis revealed that Rab5 expression was decreased by the *Dkk-3* knock-down. Morphologically, the vacuoles in the cells transfected with siR-*Dkk-3* were certainly decreased by the treatment with the macropinocytosis inhibitor or transient overexpression of Dkk-3. These conflicting results may suggest the difficulty to detect macropinocytosis by a single biomarker.

We explored a molecular mechanism leading to macropinocytosis induced by the *Dkk-3* knock-down. When the nutritional condition was poor, autophagy was facilitated in T24 cells transfected with siR-*Dkk-3*. When the extracellular nutrients were rich, T24 cells transfected with siR-*Dkk-3* underwent macropinocytosis. Autophagy is a process of self-digestion, fundamentally a cell-survival system to resist various environmental stresses, including nutrient deprivation, hypoxia and cytotoxic agents [6]. There are reports suggesting Dkk-3 involvement in the induction and inhibition of autophagy [31,32]. In the current study, it is considered that Dkk-3 knock-down induced compensatory activation of the Erk1/2 signaling pathway to survive the stress and led to the induction of autophagy or macropinocytosis according to the extracellular condition. This result indicates that the cell possesses some kind of sensor for the extracellular nutritional condition. Furthermore, the stability of the Rab5 protein was also altered according to the extracellular condition (Figure S4). When extracellular nutrients were poor, the degradation of Rab5 was suppressed in both cells transfected with non-specific control and siR-*Dkk-3*. When extracellular nutrients were rich, the degradation of Rab5 occurred during 24 h. The N-terminal residues of Rab5 are MA (methionine-alanine), which are considered to be a target of the Ac/N-end rule pathway [19]. N-end rule pathway-mediated degradation of protein has been involved in various biological processes, including cell death, and the stability of cellular fragments and their role in cell fate have been a hot topic recently [33]. Several reports have demonstrated that pro-apoptotic and anti-apoptotic proteins are degraded by the N-end rule pathway and that they regulate apoptosis induction [34,35,36]. In this study, it was considered that the degradation of the Rab5 protein by the N-end rule pathway was promoted in the nutrient-rich condition and that may have contributed to the induction of macropinocytotic cell death in T24 cells transfected with siR-*Dkk-3*.

We also examined a molecular mechanism leading to growth inhibition induced by the *Dkk-3* knock-down. Western blot analysis detected the inactivation of the Ras/Rac-1/JNK axis and activation of the p38 pathway (Figure S1) in T24 cells transfected with siR-*Dkk-3.* JNK and p38 pathways are both stress-responsive MAPK (SAPK) signal pathways that regulate apoptosis and cell-cycle. In prostate cancer, Dkk-3 overexpression causes apoptosis through the activation of JNK signaling [8]. In T24 cells, however, Dkk-3 downregulation activated the p38 pathway instead. Cell-cycle analysis demonstrated G0/G1 cell-cycle arrest and a slight increase in the population of the sub-G1 phase (apoptotic cells). Western blot analysis detected an increase in the 42-kDa fragment of PARP-1 (Figure S1). While the cleavage of PARP-1 by caspase-3 produces two specific fragments of 89 and 24 kDa that are useful hallmarks of apoptosis, cathepsin produces PARP-1 fragments of 44, 55, 62 or 74 kDa [37]. Cathepsin, a lysosomal protease, can also initiate apoptotic, autophagic and necrotic forms of cell death [38]. Activation of the p38 pathway could be a cause for cell-cycle arrest, cathepsin activation and consequent growth inhibition observed in T24 cells transfected with siR-*Dkk-3*. It is also considered that the cathepsin-initiated PARP-1 cleavage, cell death and growth inhibition would be a pathophysiology of macropinocytotic cell death. Lamp-1 glycosylation was induced in the cells transfected with siR-*Dkk-3.* Glycosylation is a post-transcriptional modification that can stabilize proteins against digestive enzymes in lysosomes. As mentioned above, it has been shown that Lamp-1 acquisition occurs according to the fusion of macropinosomes with lysosomes. Thus, *Dkk-3* knock-down induced the glycosylation of Lamp-1 to promote the digestion of macropinosomes by lysosomes and, consequently, led to the macropinocytotic catabolic cell death. Another Dkk-3 overexpressant Panc-1 cell also exhibited vesicle-rich in the cytoplasm and decreased viability after the *Dkk-3* knock-down, which was aborted by macropinocytosis inhibitor EIPA (Figure S2).

In conclusion, Dkk-3 has a novel role in the regulation of macropinocytotic and autophagic pathways, a distinct function of Dkk-3 other than a tumor suppressor. Further studies will be needed to understand the role of endogenous and secreted Dkk-3 in both physiological and pathological conditions.

## 4. Materials and Methods

### 4.1. Cell Culture, Cell Viability and Inhibitors

All human cancer cell lines used in this study were cultured in RPMI-1640 medium supplemented with 10% (*v*/*v*) heat-inactivated FBS (Sigma-Aldrich Co., St Louis, MO, USA) and 2 mM l-glutamine under an atmosphere of 95% air and 5% CO_2_ at 37 °C. The number of viable cells was determined by performing the trypan blue dye-exclusion test. Cycloheximide (Sigma-Aldrich Co.) was used to inhibit protein synthesis at the translational step. MG132 (Sigma-Aldrich Co.) was used to inhibit proteasomal degradation of protein. 3-methyladenine (3-MA) (Calbiochem, Merck Millipore, Darmstadt, Germany) and 5-(*N*-ethyl-*N*-isopropyl) amiloride (EIPA) (Sigma-Aldrich Co.) were used to inhibit autophagy and macropinocytosis, respectively.

### 4.2. Transfection with miR-92a or Short-Interfering RNA for Dkk-3

T24 and Panc-1 cells were seeded in six-well plates at a concentration of 0.5 × 10^5^ per well (10%–30% confluence) on the day before the transfection. The mature type of miR-92a (mirVana miRNA mimic; Ambion, Foster City, CA, USA) or short-interfering RNA (siRNA) for *Dkk-3* (siR-*Dkk-3*; Invitrogen, Carlsbad, CA, USA) was used for the transfection of the cells, which was achieved by using cationic liposomes, Lipofectamine RNAiMAX (Invitrogen), according to the manufacturer’s Lipofection protocol. When this reagent was used in the transfection of adherent cells, 60%–80% of the cells are usually transfected in our laboratory. The nonspecific control miRNA (HSS, Hokkaido, Japan) sequence was 5′-GUAGGAGUAGUGAAAGGCC-3′, which was used as a control for nonspecific effects. The sequence of the mature type of miR-92a used in this study was 5′-UAUUGCACUUGUCCCGGCCUGU-3′ and those of siR-*Dkk-3* were 5′-GAUGAGUAUGAAGUUGGCAGCUUCA-3′ and 5′-CCCTCTTTGGCAGTTGCATTAGTAA-3′. The effects manifested by the introduction of siR-*Dkk-3* or miR-92a into the cells were assessed at selected time points after the transfection.

### 4.3. RNA Extraction and Real-Time Reverse Transcription

Total RNA was isolated from cultured cells or tumor tissues by using a NucleoSpin miRNA isolation kit (TakaRa, Otsu, Japan). RNA concentration and purity were assessed by UV spectrophotometry. RNA integrity was checked by formaldehyde gel electrophoresis. For the determination of the expression levels of mRNAs, total RNA was reverse-transcribed with PrimeScript RT reagent Kit (TakaRa). The quantitative reverse transcription-polymerase chain reaction (qRT-PCR) was performed with primers specific for Dkk-3 by using THUNDERBIRD SYBR qPCR mix (TOYOBO Co., Ltd., Osaka, Japan). The primers for Dkk-3 and glyceraldehyde 3-phosphate dehydrogenase (GAPDH) were given as follows: Dkk-3-sense, 5′-TTCGGGTAGTGGAAAACCAG-3′, and Dkk-3-antisense, 5′-CAGCAGCTCGAATTTCTTCC-3′; GAPDH-sense, 5′-CTCAGACGGCAGGTCAGGTCCACC-3′, and GAPDH-antisense, 5′-CCACCCATGGCAAATTCCATGGCA-3′. GAPDH was used as an internal control. All reactions were run in triplicate. The relative expression levels were calculated by the ΔΔ*C*_t_ method.

### 4.4. Western Blot Analysis

Protein extraction and Western blotting experiments were performed as described previously. Primary antibodies used were as follows: antibodies against Dkk-3 (Santa Cruz, Santa Cruz, CA, USA); phospho-JNK, JNK, Cyclin D1, phospho-Erk1/2, Erk1/2, CDK4, CDK6, PAK4, Ras, Rac-1, Rab5, phospho-p38, p38 and LC3B (Cell Signaling Technology, Danvers, MA, USA); Sirt-1 and Lamp-1 (Abcam, Cambridge, UK); and anti-β-actin (Sigma-Aldrich Co.). HRP-conjugated horse anti-mouse and goat anti-rabbit IgG antibody (Cell Signaling Technology) were used as secondary antibodies.

### 4.5. Immunofluorescence Staining

T24 cells were seeded into the wells of a Lab-Tek™ II Chamber Slide System (Thermo Fisher Scientific Inc., Waltham, MA, USA) at a concentration of 1.0 × 10^5^ cells/well the day before transfection. At 72 h after the transfection with a non-specific control, siR-*Dkk-3* (1 or 2 nM) or miR-92a (10 or 20 nM) was fixed by 4% formaldehyde for 10 min, and the chambers were washed by PBS for 15 min. T24 cells were stained with anti-Lamp-1 antibody (Abcam) and anti-rabbit IgG conjugated with Alexa Fluor 488 (Molecular Probes, Eugene, OR, USA) for immunofluorescence localization of Lamp-1. T24 cells were also stained with Hoechst33342 (5 μg/mL) and anti-phalloidin antibody (Cytoskeleton Inc., Denver, CO, USA) according to the immunofluorescence protocol of Cell Signaling Technology. Labeled cells were observed with a Biorevo fluorescence microscope (Keyence, Osaka, Japan). In the experiment of nutrient deprivation, T24 cells were transfected with non-specific control or siR-*Dkk-3* in three different conditions. Cells in one condition were deprived of FBS for 6 h after transfection. Cells in another condition were cultured with the FBS-added medium for 6 h after transfection, and then, the medium was replaced with FBS-free medium at 24 h after transfection. Cells in the other condition were cultured within the FBS-added medium after transfection. Cells were stained at 48 h after transfection as described above.

### 4.6. Cell Cycle Analysis

Quantification of cellular DNA content at 48 h after transfection with non-specific control, miR-92a (10, 20 or 40 nM) or siR-*Dkk-3* (1, 2 or 5 nM) was determined by using a cytometer. Briefly, the cells were harvested and fixed with 70% cold ethanol at −20 °C overnight. The fixed cells were washed twice with PBS, resuspended in 100 μL PBS-based propidium iodide solution containing 0.1% Triton X-100 (Wako Pure Chemical Industries, Ltd., Osaka, Japan), 0.2 mg/mL RNase A (Invitrogen) and 20 μg/mL propidium iodide (Invitrogen) and incubated for 30 min at room temperature protected from the light. The DNA content in the cells was analyzed through the cytometer (The Tali^®^ Image-Based Cytometer, Invitrogen).

### 4.7. Electron Microscope Observation

T24 cells were harvested at selected time points after the transfection, fixed for 30 min in 4% paraformaldehyde and 25% glutaraldehyde in 0.1 M phosphate buffer (pH 7.0) and post-fixed in 1% osmium tetraoxide for 30 min. The cells were progressively dehydrated by passage through a 10% graded series of 50%–100% ethanol and then cleared in QY-1 (Nissin EM, Tokyo, Japan). They were then embedded in Epon 812 resin (TAAB Laboratories Equipment, Reading, UK); subsequently, thin sections (70 nm thickness) were cut, stained with uranyl acetate and lead citrate and then examined by transmission electron microscopy using an Hitachi-7650 (Hitachi, Tokyo, Japan). The area of macropinocytotic vesicles in each cell was measured by ImageJ (Wayne Rasband NIH, Bethesda, MD, USA). Statistical analysis was done by Student’s *t*-test.

### 4.8. 3D Spheroid Colorimetric Proliferation/Viability Assay

The 3D spheroid colorimetric proliferation/viability assay was performed according to the manufacturer’s protocol of a Cultrex 3-D spheroid Colorimetric Proliferation/Viability Assay Reagent Kit (Trevigen, Inc., Gaithersburg, MD, USA). Briefly, T24 cells were transfected with non-specific control or siR-*Dkk-3* (1 or 2 nM) and harvested at 24 h after transfection. The cells were then resuspended in spheroid formation ECM at a concentration of 3000 cells/50 μL and seeded in the 3D Culture Qualified 96 Well Spheroid Formation Plate at 50 μL/well. The plate was centrifuged at 200× *g* for 3 min at room temperature and incubated at 37 °C for 72 h to promote spheroid formation. After 72 h, the spheroids were photographed with the microscope (CKX41, Olympus, Tokyo, Japan), and the images were analyzed using image analysis software to measure changes in the area of the structures to determine the extent of 3D culture spheroid expansion for each sample. After that, the MTT assay was also performed according to the manufacturer’s protocol. Absorbance was read at 570 nm by the microplate reader (iMark microplate reader, Bio-Rad Laboratories Inc., Hercules, CA, USA).

### 4.9. Dkk-3 Overexpression

The eukaryote Dkk-3 expression vector was generated by inserting the open reading frame of Dkk-3 cDNA into the *Sgf* I and *Pme* I site of the pF5A CMV-neo Flexi vector (Promega, Madison, WI, USA). Dkk-3 was transiently overexpressed in T24 cells achieved by the transfection of the expression vector (0.5 μg/mL) using Lipofectamine 2000 (Invitrogen).

### 4.10. Statistics

Each examination was performed in triplicate. The cell count analysis and the expression levels of mRNAs in the cells transfected with miR-92a and those transfected with nonspecific control miRNA were compared by using Student’s *t*-test. A *p*-value less than 0.05 was considered to be statistically significant.

## Supplementary Materials

Supplementary materials can be found at www.mdpi.com/1422-0067/17/11/1846/s1.
