# Supplementary material for: A Novel Role of Dickkopf-Related Protein 3 in Macropinocytosis in Human Bladder Cancer T24 Cells
Source: Int J Mol Sci. 2016 Nov 5;17(11):1846. doi: 10.3390/ijms17111846 (PMC5133846; doi:10.3390/ijms17111846)
Supplement: Supplementary file 1 [file ijms-17-01846-s001.pdf]

Supplementary Materials: A Novel Role of Dickkopf-Related Protein in Macropinocytosis in Human Bladder Cancer T24 Cells

Nonoka Tsujimura, Nami O. Yamada, Yuki Kuranaga, Minami Kumazaki, Haruka Shinohara, Kohei Taniguchi and Yukihiro Akao

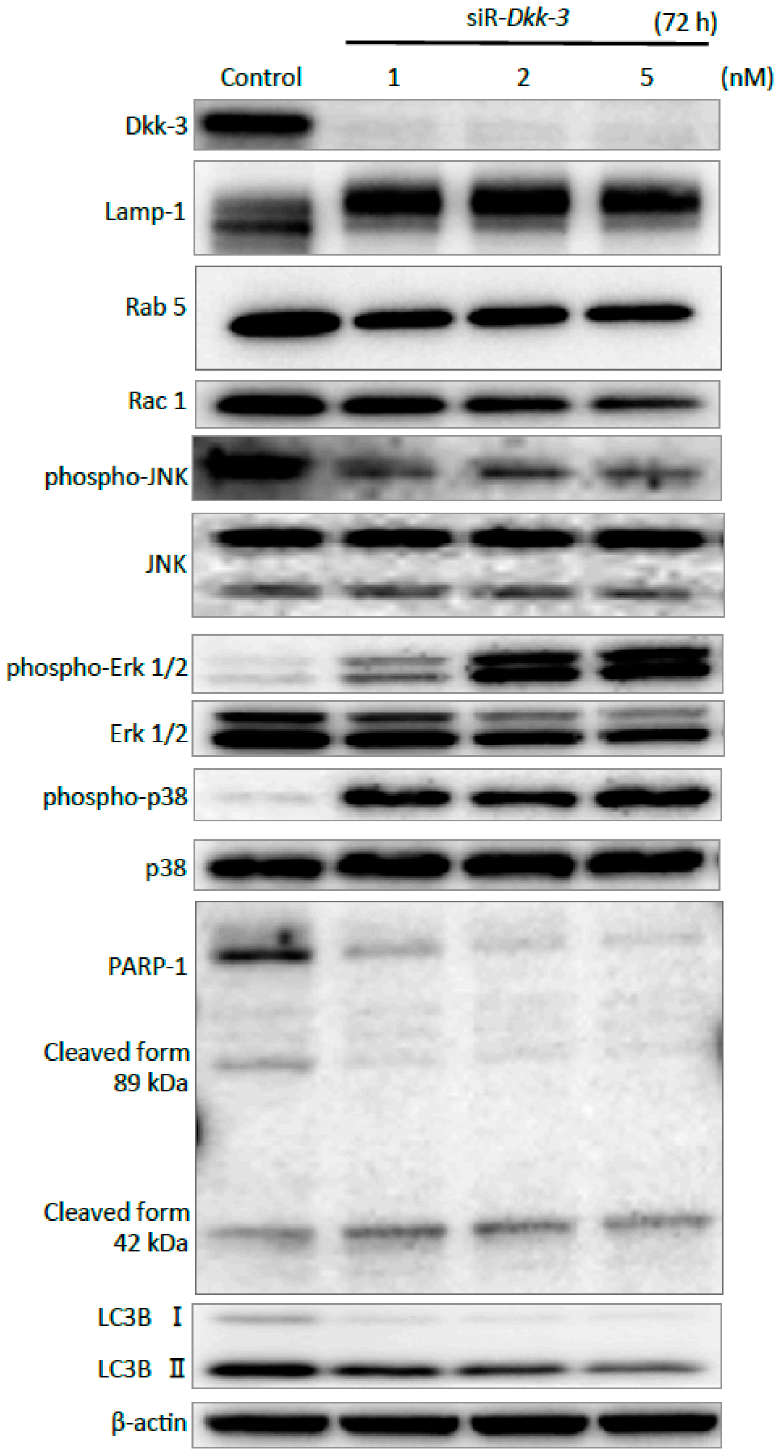

Figure S1. Expression profiles of autophagy, apoptosis or macropinocytosis related proteins in T24 cells at 72 h after transfection with non-specific siRNA or siR-*Dkk-3*.

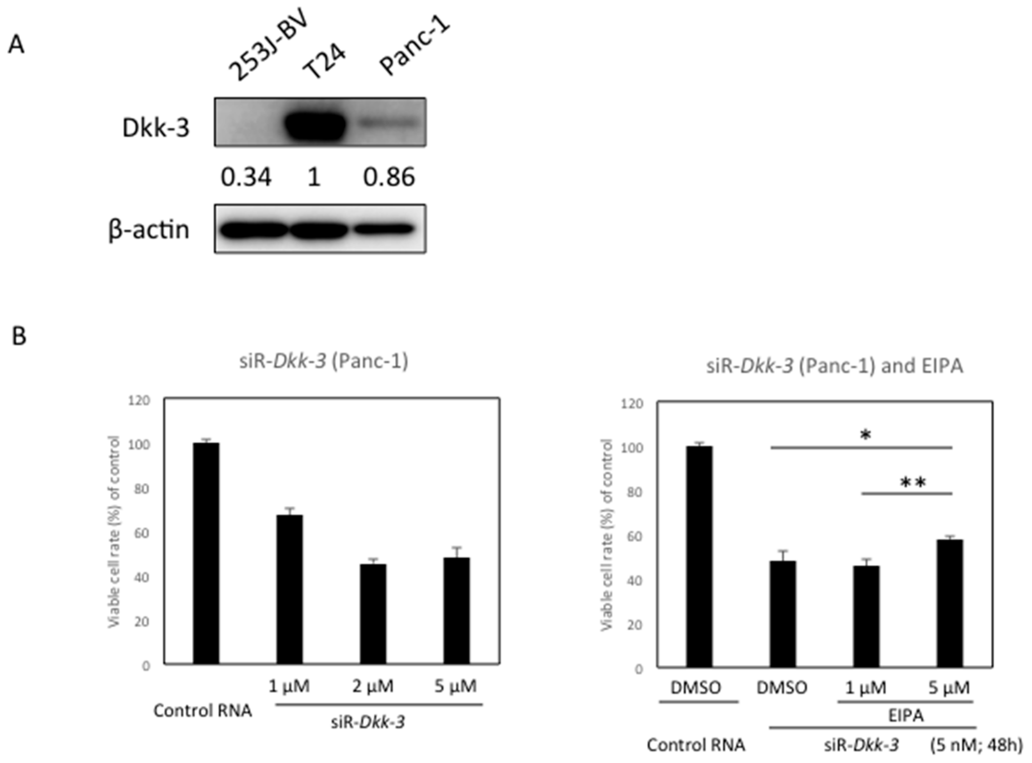

Figure S2. Growth inhibitory effects of *Dkk-3* knock-down in Panc-1 cells. (**A**,**B**) Dkk-3 expression and Cell viability in Panc-1 cells at 48 h after transfection with non-specific siRNA or siR-*Dkk-3* (1, 2, or 5 nM), which was canceled in part by the co-treatment with EIPA. The Dkk-3 expression levels were estimated by the densitometric analysis. The *p*-values in (**B**) are indicated as follows: * $p < 0.05$ and ** $p < 0.01$.

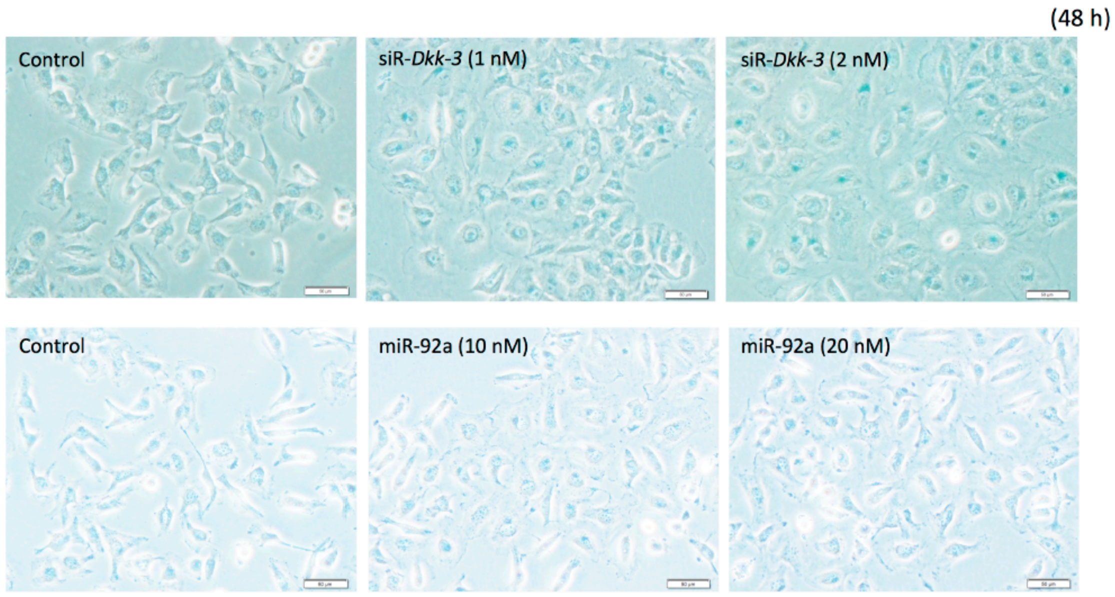

Figure S3. Staining for senescence-associated β-galactosidase in T24 cells at 48 h after transfection with non-specific siRNA, siR-*Dkk-3* or miR-92a. Scale bars, 50 µm.

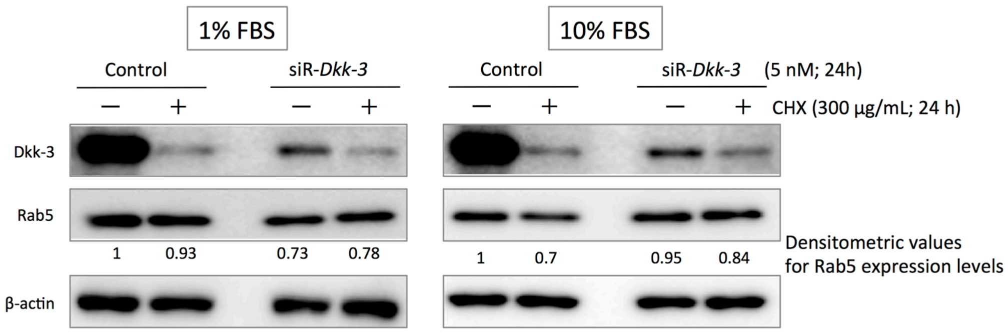

Figure S4. Stability of Rab5 protein in T24 cells transfected with non-specific siRNA or siR-*Dkk-3* (5 nM) in different nutritional conditions (1% FBS- or 10% FBS-containing medium). Cells were also treated with CHX (300 μg/mL) at the time of transfection.
